# A noise model for the evaluation of defect states in solar cells

**DOI:** 10.1038/srep29685

**Published:** 2016-07-14

**Authors:** G. Landi, C. Barone, C. Mauro, H. C. Neitzert, S. Pagano

**Affiliations:** 1 Dipartimento di Ingegneria Industriale, Università di Salerno, I-84084 Fisciano, Salerno, Italy; 2 Dipartimento di Fisica “E.R. Caianiello”, Università di Salerno, I-84084 Fisciano, Salerno, Italy; 3 CNR-SPIN, UOS di Salerno, I-84084 Fisciano, Salerno, Italy

## Abstract

A theoretical model, combining trapping/detrapping and recombination mechanisms, is formulated to explain the origin of random current fluctuations in silicon-based solar cells. In this framework, the comparison between dark and photo-induced noise allows the determination of important electronic parameters of the defect states. A detailed analysis of the electric noise, at different temperatures and for different illumination levels, is reported for crystalline silicon-based solar cells, in the pristine form and after artificial degradation with high energy protons. The evolution of the dominating defect properties is studied through noise spectroscopy.

The study of fluctuation mechanisms is a powerful and non-destructive spectroscopic tool, useful to provide information on the dynamic behaviours and the kinetic processes of the charge carriers in condensed matter^1^. Low-frequency electric noise analysis was proposed, twenty years ago, to determine quality of solar cells and photovoltaic modules^2^,^3^,^4^. Subsequently, it was used for the reliability estimation of photovoltaic devices related to microplasma detection^5^, and of iron disilicide heterojunction solar cells^6^. More recently, the same tool was employed to evaluate transport properties and degradation processes in several other systems, such as organic light-emitting diodes^7^, polymer:fullerene solar cells^8^,^9^, bulk homogeneous semiconductors^10^,^11^, and polymer/carbon nanotube composites^12^.

In semiconducting devices, often a 1/f-type noise spectrum is observed. Semi-empirical models have been formulated to correlate the current noise to mobility fluctuations^13^,^14^, to the number fluctuation of free charge carriers within the space charge region^15^, or to the semiconductor surface^16^. However, Hsu^17^ and van der Ziel^18^ were the first to relate the 1/f-type noise in the metal-oxide-semiconductor system and in p-n junctions to the fluctuation of the defect state population. In this work, it is shown that low-frequency noise spectroscopy can be used as a sensitive tool for the investigation of the properties of these defect states. In particular, a noise model is proposed to interpret experimental data in the case of pristine and artificially degraded silicon-based photovoltaic devices. The reported results show that the formation of the defects, activated under illumination or charge carrier injection, is related to long-term degradation of the solar cells. Noise analysis can also provide interesting information on radiation damage, and can be used for a detailed temperature-dependent electrical characterization of the charge carrier capture/emission and recombination kinetics. This aspect represents an advantage of the fluctuation spectroscopic technique, which gives the possibility to directly evaluate the cell health state.

## Noise Model of Solar Cells

**Figure 1:**
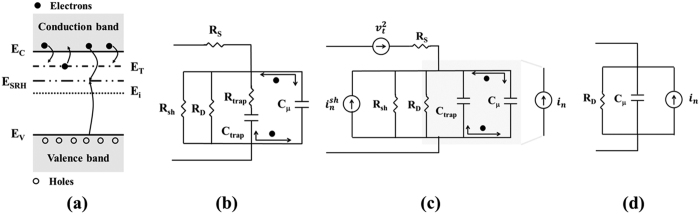
Solar cell noise equivalent circuit. (**a**) Energy-band diagram of the silicon material representing the carrier recombination and trapping processes. (**b**) ac electrical equivalent circuit of the solar cell. (**c**) Equivalent circuit including noise sources. (**d**) Simplified version.

Fluctuations in p-n junction solar cells strongly depend on the diode geometry^13^, on the photogeneration processes, and on the bias voltage^15^. In presence of defect states, the trapping and recombination mechanism of the charge carriers produces current fluctuations, that can be the main noise source^11^. The amplitude of the random current fluctuations *Var* [*I*] is, therefore, related to the occupation probability *f*_*trap*_ of the traps. Moreover, the recombination pathways from the conduction band to the valence band generate a drop of the minority charge carriers stored in the base material, and this effect also influences *Var* [*I*]. The main contribution of the traps can be modeled by two energy levels, which act as trapping (*E*_*T*_) and recombination (*E*_*SRH*_) centers. These centers are located above the intrinsic Fermi level *E*_*i*_ (see Fig. 1(a)) and are responsible for the transitions between the defect states and the conduction *E*_*C*_ and valence *E*_*V*_ bands. Under injected or photogenerated charge carriers, the *E*_*T*_ energetic states, having density *N*_*T*_, are able to capture and emit electrons from the conduction band. At the same time, the *E*_*SRH*_ states, having density *N*_*SRH*_, work as Shockley-Read-Hall (SRH) type recombination centers with an associated lifetime *τ*_*SRH*_^19^.

At low frequencies and in forward bias condition, the solar cell ac equivalent circuit, representing the SRH recombination processes, is simply composed by the parallel connection of a differential resistance *R*_*D*_ and a capacitance *C*_*μ*_, representing the contribution of the minority charge carriers into the base material^8^. The fluctuating traps with energy level *E*_*T*_ can be modeled by a series *RC* circuit in parallel to *C*_*μ*_^20^, as shown in Fig. 1(b) where the cell shunt *R*_*sh*_ and series *R*_*S*_ resistances are also displayed. Since for a typical solar cell 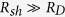, its effect on current transport can be considered negligible. Therefore, the recombination phenomena, associated to the defect states, can be well described by the time constant *τ*_*eff*_ = *R*_*D*_*C*_*μ*_. The density of empty traps into the base material introduces a capacitance contribution *C*_*trap*_, which is related to the variation of *f*_*trap*_ with respect to the quasi-Fermi level under nonequilibrium condition (charge carrier injection). The resistance *R*_*trap*_, instead, represents the kinetic factor of the traps^20^, which are governed by a characteristic trapping/detrapping time *τ*_*trap*_ = *R*_*trap*_*C*_*trap*_. In the frequency region below (2*πτ*_*trap*_)^−1^, the capacitive contribution of the fluctuating trap states becomes significant and the noise spectroscopy is able to follow the charge carrier transfer from the conduction band to empty traps and viceversa (see Fig. 1(c)). This mechanism leads to fluctuations in the number of charge carriers and contributes, therefore, to a current fluctuation that can be measured by the external contacts. The effect of these traps can be modeled as a current-noise source *i*_*n*_, which represents the charge carriers transfer between *C*_*trap*_ and *C*_*μ*_ (see Fig. 1(d)). Thermal noise contributions 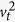 and 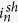, related to *R*_*S*_ and *R*_*sh*_, respectively, are usually negligible for solar cells, as well as the shot noise present in p-n junctions^1^. As a consequence, the solar cell equivalent noise circuit can be assumed as simply composed by the parallel connection of *C*_*μ*_, *R*_*D*_, and *i*_*n*_, as shown in Fig. 1(d). The noise is generated by the change in the number of carriers, that are typically present in the cell, due to the dc bias or light absorption. A characteristic 1/f frequency spectrum is observed, with the presence of a cut-off frequency evaluated as^8^ *f*_*x*_ = (2*πτ*_*eff*_)^−1^.

In this framework, the amplitude *Var* [*I*] of the 1/f noise component can be defined as the sum of a dark (*Var* [*I*_*dark*_]) and a photo-induced (*Var* [*I*_*ph*_]) contribution. Under dark condition and on forward biasing of the solar cell, the *Var* [*I*_*dark*_] value is influenced by the emission rates *e*_*n*,*p*_ (dimensions of s^−1^) and volumetric capture coefficients per unit time *c*_*n*,*p*_ (dimensions of cm^3^ s^−1^) for the electron and hole traps^19^. When the cell is illuminated, these capture coefficients and emission rates change, as well as the density of filled traps^21^. As a consequence, the process of carrier photogeneration increases the trap filling until a saturation occurs, meaning that all traps have been activated from the noise point of view. In this situation, the *Var* [*I*_*ph*_] contribution is dominant, resulting larger than the dark background noise.

The photogenerated current can be expressed as^22^


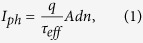


where *q* is the elementary charge, *n* is the average excess carrier density, and *A* and *d* are the sample area and thickness, respectively. By taking into account the SRH statistics, the resulting fraction of the filled fluctuating traps is^19^


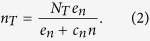


Moreover, the fraction of the filled recombination centers is^19^


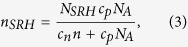


where *N*_*A*_ is the base doping concentration. In equation (1) the only fluctuating term is related to *n*, therefore, under the assumption of a binomial probability distribution, *Var* [*I*_*ph*_] can be expressed as^1^


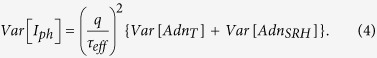


Substituting equations (2) and (3) into equation (4) gives


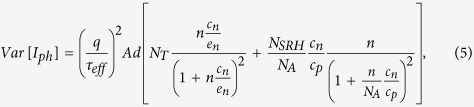


where *c*_*n*_/*c*_*p*_ = *k* is the symmetric ratio^23^.

By defining


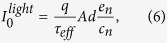


and


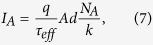


equation (5) can be rewritten as


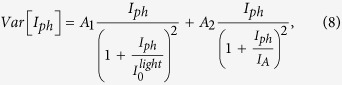


where


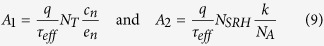


represent the amplitudes of the current fluctuations related to the trapping and recombination mechanisms, respectively. Here, 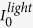 is interpreted as the threshold current at which the noise level is almost saturated, and *I*_*A*_ takes into account the influence of the recombination centers.

## Experimental Results and Discussion

The proposed noise model, equations (8) and (9), has been used to analyze experimental results from Si-based solar cells, in their pristine form and after a sequence of proton irradiations, in order to generate an increasing number of traps.

### Pristine Si-based solar cells

**Figure 2:**
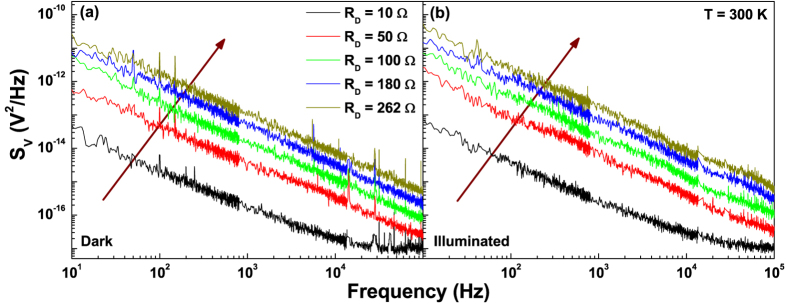
Voltage-spectral density of pristine silicon solar cells. The frequency dependence of *S*_*V*_, at 300 K and for several differential resistance *R*_*D*_ values, is shown in dark (**a**) and under illumination with 18 mW/cm^2^ (**b**). The arrows indicate increasing *R*_*D*_.

The study of the fluctuation spectroscopy requires the acquisition of the voltage-spectral density *S*_*V*_ generated by the device in various operation conditions. In Fig. 2, the low-frequency *S*_*V*_ dependence of a pristine Si-based solar cell is shown at a temperature of 300 K and for different bias points, corresponding to several differential resistance values. Apart from a number of peaks at definite frequencies related to external noise sources, a large 1/f component is evident, followed by a constant amplitude spectrum at higher frequencies. This behaviour has been observed in the whole investigated temperature range, both in dark, see Fig. 2(a), and under illumination, see Fig. 2(b). The measured noise can be well reproduced by^1^


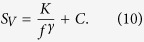


All the coefficients in equation (10) have been experimentally evaluated with a high level of accuracy and a statistical error lower than 2%. In particular, *K* is the voltage-noise amplitude, *γ* = (1.18 ± 0.02) is the frequency exponent, and *C* = 1.04 × 10^−17^ V^2^/Hz is a frequency-independent noise component, due to the readout electronics, that can be neglected.

**Figure 3:**
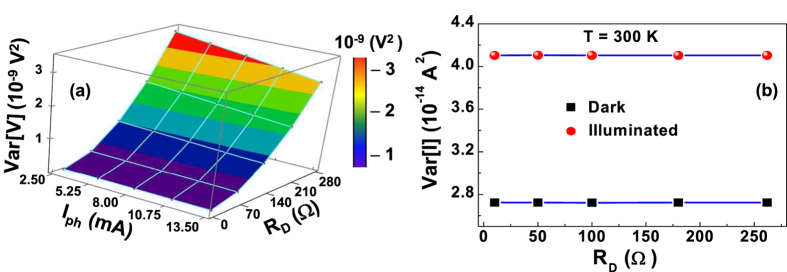
Voltage- and current-noise amplitudes. (**a**) Dependence of the 1/f voltage-noise amplitude on the differential resistance at 300 K and on photocurrent. (**b**) Dependence of the current fluctuations amplitude on the differential resistance at room temperature, under illumination at 18 mW/cm^2^ (circles) and under dark condition (squares).

By examining in details the 1/f noise, interesting information on the conduction mechanisms and the dynamics of fluctuations can be extracted. The variance of the voltage can be computed from the experimental data as^1^


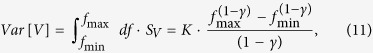


where the frequency interval [ *f*_min_, *f*_max_] = [1, 100000] Hz is the experimental bandwidth, and *γ* ≠ 1. The *Var* [*V*] is characterized by a quadratic dependence on *R*_*D*_ at all illumination and temperature conditions, as shown in Fig. 3(a) for 300 K. This result clearly indicates that current fluctuations are the dominant noise source in the photovoltaic system. Their amplitude being given by^1^


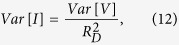


and is independent on *R*_*D*_, as evidenced in Fig. 3(b).

**Figure 4:**
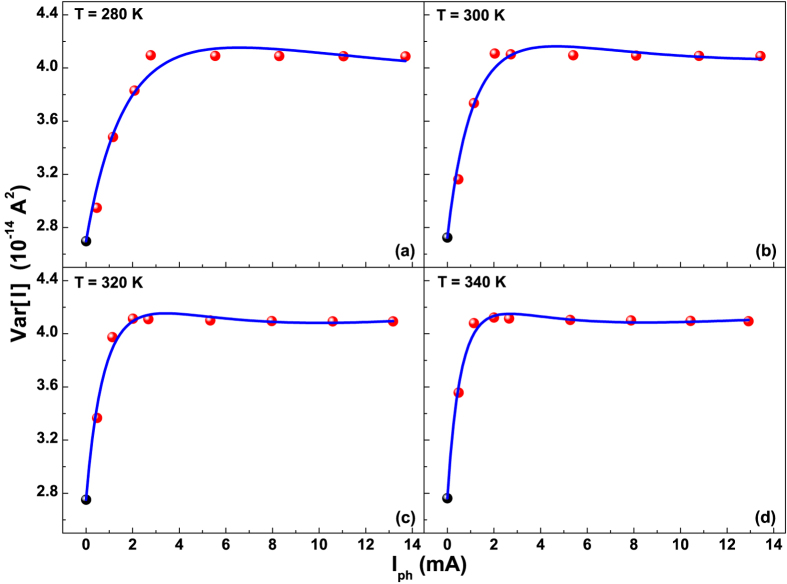
Photo-induced noise fluctuations between 280 and 340 K. Photocurrent dependence of the current fluctuations amplitude at: 280 K (**a**), 300 K (**b**), 320 K (**c**), and 340 K (**d**). The best fitting curves, using equation (8), are shown as blue solid lines. The values for *I*_*ph*_ = 0 are the noise in dark (black dots).

Due to the independence of *Var* [*I*] on *R*_*D*_, the photo-induced fluctuations amplitude has been analyzed at a fixed bias condition of *R*_*D*_ = 10 Ω, and is shown as current noise in Fig. 4 at four different temperatures between 280 and 340 K. The theoretical model of equation (8) has been used to fit the experimental data and is depicted in Fig. 4 as blue solid curves. Under the typical low-level injection operating conditions of most silicon solar cells^24^, the SRH recombination is the dominant mechanism which affects the minority charge carrier lifetime. Therefore, it can be assumed that 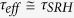. Furthermore, for the investigated devices, in the framework of the SRH model, it is possible to express 
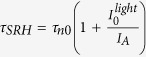
, with *τ*_*n*0_ = (*c*_*n*_*N*_*SRH*_)^−1^. Being 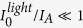, varying from (2.04 ± 0.03) × 10^−2^ at 280 K to (3.90 ± 0.06) × 10^−2^ at 340 K, it results that 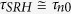. Since *τ*_*n*0_ is weakly temperature-dependent below 340 K, as already verified for boron-doped Czochralski (Cz) silicon cells^23^, similar to those analyzed here, the value of *τ*_*eff*_ can be assumed constant. It has been obtained from ref. 25, where photoelectric measurements of the same devices are reported at room temperature. Here, it is worth noting that the effective lifetime can be directly estimated from the noise spectra, when the cut-off frequency occurs within the experimental frequency bandwidth^8^. Otherwise, alternative techniques have to be used for the evaluation of *τ*_*eff*_^26^.

**Figure 5:**
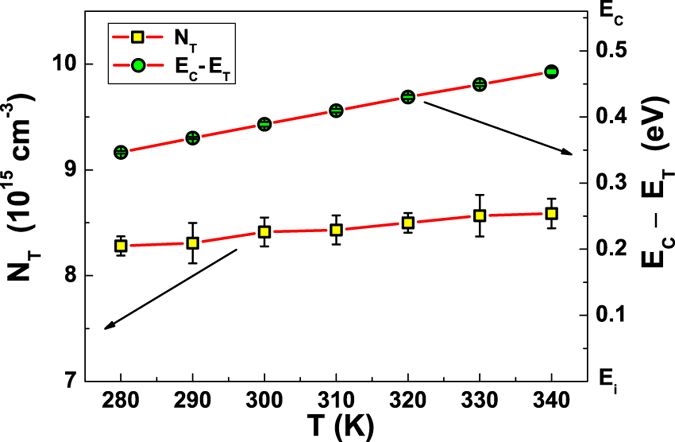
Temperature dependence of the defect states parameters. The fluctuating trap density *N*_*T*_ is shown as a function of temperature on the left y-axis (yellow squares), while the energy depth of the traps *E*_*C*_ − *E*_*T*_ is shown on the right y-axis (green circles). The right scale refers to the upper half *E*_*C*_ − *E*_*i*_ of the bandgap.

From the best values of the fitting coefficients 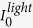, *I*_*A*_, *A*_1_, and *A*_2_ (see Supplementary Table S1 for details), by using equations (6), (7) and (9), it is straightforward to compute the most important defect states parameters. In particular, for low illumination level, *I*_*A*_ and *A*_2_ are such that the main contribution to current noise comes from the first part of equation (8), where the number of fluctuating traps *N*_*T*_ plays an important role. The temperature dependence of *N*_*T*_ is reported in Fig. 5 (left axis), showing that it does not change within the experimental errors in the whole analyzed temperature range. It is worth noting that, the average value of *N*_*T*_ = (8.4 ± 0.1) × 10^15^ cm^−3^ is close to the doping concentration 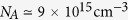. This is consistent with the formation of unstable boron-oxygen defects. Moreover, the energy depth of the traps below the conduction band can be estimated from


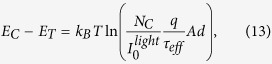


where *k*_*B*_ is the Boltzmann constant, *T* the temperature, and *N*_*C*_ the effective density of states in the conduction band^22^. The temperature dependence of the trap energy is shown in Fig. 5 (right axis), from which an average value of *E*_*C*_ − *E*_*T*_ = (0.41 ± 0.04) eV can be evaluated. Again, the estimations of *E*_*C*_ − *E*_*T*_ and of the symmetric ratio *k* = (30.9 ± 0.8) at 300 K are in good agreement with what found in literature for a metastable boron-oxygen defect in Cz grown silicon, where *k* = 6.2 − 33.6 and *E*_*C*_ − *E*_*T*_ = 0.39 − 0.46 eV are reported^23^.

### Proton irradiated Si-based solar cells

**Figure 6:**
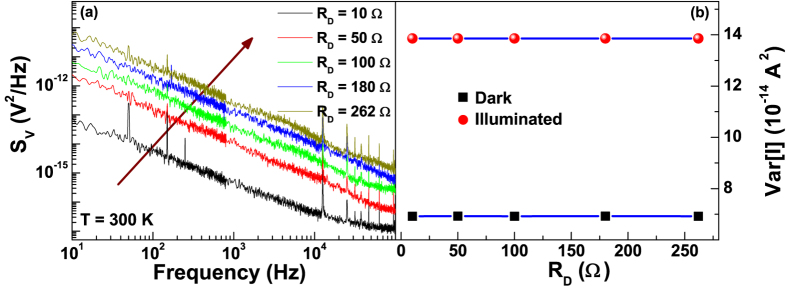
Voltage and current noise for irradiated devices. (**a**) The frequency dependence of the voltage-spectral density, at 300 K and several differential resistances, is shown for a proton irradiated silicon solar cell with a fluence of 5 × 10^12^ protons/cm^2^. (**b**) The independence of the 1/f current-noise amplitude on the differential resistance is reported both in dark (squares) and under illumination at 18 mW/cm^2^ (circles).

As for pristine devices, the same frequency dependence of *S*_*V*_ is observed for all the silicon solar cells irradiated with different proton fluences. In particular, in Fig. 6(a) it is shown, at a temperature of 300 K and in dark condition, the presence of a large 1/f noise component followed by a constant amplitude spectrum at higher frequencies. This 1/f contribution is characterized by a quadratic dependence on the differential resistance *R*_*D*_. As a consequence, the current-noise amplitude *Var* [*I*] can be computed from equation (12) and does not depend on *R*_*D*_, as clearly displayed in Fig. 6(b). The overall behaviour results to be completely independent on temperature, proton irradiation, and light intensity, thus indicating again current fluctuations as the dominant noise source^1^.

**Figure 7:**
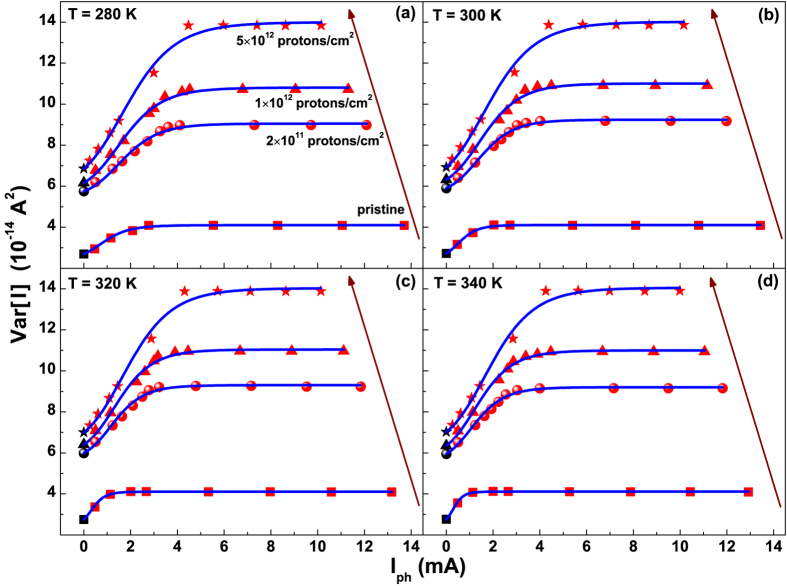
Photo-induced noise fluctuations for pristine and irradiated devices. Dependence of the current fluctuations amplitude on the photocurrent for temperatures from 280 to 340 K. The experimental data points and the best fitting curves, using equation (8), are shown for pristine and irradiated samples. The arrows indicate increasing proton fluences.

In the whole investigated temperature range and for all the proton irradiation fluences, the quantity *Var* [*I*] is characterized by the same photocurrent dependence already observed for pristine devices. This is shown in Fig. 7. The theoretical model of equation (8) has been applied to the artificially degraded silicon solar cells. Again a good agreement is found between the best fitting curves obtained from equation (8) (blue solid curves in Fig. 7) and the experimental data (full symbols in Fig. 7).

**Figure 8:**
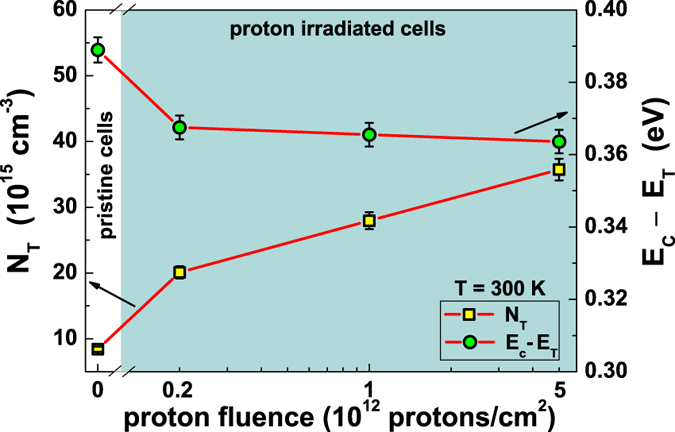
Degradation effect on the defect states parameters at 300 K. The proton fluence dependence, at room temperature, is shown for the fluctuating trap density *N*_*T*_ (left y-axis and yellow squares), and for the energy depth of the traps *E*_*C*_ − *E*_*T*_ (right y-axis and green circles).

The dependence on irradiation level of the defect states parameters, extracted from the noise analysis, allows to identify the effect of radiation exposure, and the corresponding damage of the cells, which is also associated to a decrease of the energy conversion efficiency^25^. In Fig. 8 (left axis), is shown at 300 K the density *N*_*T*_ of active traps contributing significantly to the noise signal, that increases with the fluence of proton irradiation. This behaviour gives a direct indication of the presence of additional degradation processes in the photovoltaic device, also confirmed by the increase of the dark background noise amplitude. It means that the defects created by damaging the samples have the same role in the fluctuation mechanisms both in dark and under illumination. At the same time, a weak decrease of the trap energy depth is displayed in Fig. 8 (right axis) at room temperature. As reported in literature, after proton degradation, the dominant radiation-induced defect in boron-doped Cz grown silicon is related to the donor-like defects^27^. Such a defect state, located at *E*_*C*_ − 0.18 eV, acts as a minority carrier trap and influences the trapping/detrapping. This indicates that under low charge carrier injection, as the case here considered, the fluctuating traps closer to the conduction band produce the main contribution to the measured current noise.

The observed effects on *N*_*T*_ and on *E*_*C*_ − *E*_*T*_ are consistent with the reported long wavelength decrease of the external quantum efficiency, due to the increase of recombination centers density caused by proton irradiation^25^,^28^. Moreover, the most striking feature of Fig. 8 is an abrupt change evident after the first dose of radiation. This finding suggests that the major disturbance of the structure, leading to a noise enhancement, seems to occur at the beginning of radiation exposure. The values reported in Table 1 are in agreement with this experimental evidence. In particular, the simultaneous decrease of the relevant solar cell photoelectric parameters and the increase of trap density seem to be directly related to an overall reduction in the power conversion efficiency.

## Conclusions

It has been shown that noise spectroscopy is a sensitive tool for the electrical temperature-dependent characterization of electronic defects in photovoltaic devices. A theoretical model has been formulated to explain the nature of current fluctuations in solar cells. The applicability of the proposed model has been verified on pristine and artificially degraded Si-based devices. Distinct differences between dark and photo-induced noise have been found and interpreted in terms of a Shockley-Read-Hall theory. In particular, a combination of trapping/detrapping related processes and charge carrier recombination phenomena has been considered to explain the current fluctuation mechanisms. Characteristic parameters of the dominant defect centers, as extracted by noise analysis, have been compared with literature data. In pristine cells, the energetic position and the symmetric ratio are in good agreement with the values obtained by using alternative techniques, and well consistent with the boron-oxygen complex defect. On the other hand, in degraded devices, the total trap density increases with the fluence of proton irradiation and seems to be directly related to a decrease of the power conversion efficiency. Application of this noise model to other photovoltaic materials, such as organic and perovskite compounds, is currently in progress.

## Methods

The solar cells, used to test the validity of the proposed electric noise model, were realized by SOLARTEC, type “SC2140-Z8-24”. The investigated area *A* of the photovoltaic devices was 1 cm^2^ with a wafer thickness *d* of 320 *μ*m. The initial efficiency of pristine samples was measured to be 15% under AM 1.5 G conditions, with a short-circuit current *I*_*sc*_ = 37.45 mA, open-circuit voltage *V*_*oc*_ = 581 mV, and fill factor *FF* = 68.96% (see ref. 25 for details). Degradation effects were artificially induced on the devices with proton irradiation, realized at the Helmholtz-Zentrum Berlin für Materialien und Energie (Germany), with a proton energy of 65 MeV in air. A homogeneous defect distribution, with this energy value, was verified by using a SRIM (Stopping and Range of Ions in Matter) code. Specific information on the analysis performed can be still found in ref. 25. The samples were irradiated with three different proton fluences: 2 × 10^11^ protons/cm^2^, 1 × 10^12^ protons/cm^2^, and 5 × 10^12^ protons/cm^2^.

All the measurements were carried out by varying the temperature from 280 to 340 K with a thermoelectric cooler, and by stabilizing it with a computer-controlled PID loop to better than 0.1 K. A commercial cool white Light Emitting Diode (LED) “KLC8 Edixeon K series”, from Edison Opto, was used as light source. This choice of adopting a LED source was done in order to have a low-noise source and to discriminate intrinsic photo-induced mechanisms from extrinsic ones, potentially related to the complex electronics of the solar simulator.

The setup for the electrical transport and noise characterizations was arranged in order to minimize the presence of spurious components in the measured spectral traces. A low-noise Keithley dc current source was used for the biasing of the samples. The dc voltage drop was recorded with a digital multimeter, while the ac voltage signal was amplified with a low-noise PAR5113 preamplifier and analyzed by a dynamic signal analyzer HP35670A. A specific procedure, based on a sequence of four-probe and two-probe noise measurements^29^, allowed the estimation and removing of contact noise contributions, a possible source of artifacts in this type of experiments. External noise contributions, such as the photon number fluctuation, light source bias circuit noise, and light source emission noise, were also experimentally evaluated and their influence was systematically excluded (see for details page 3 in Supplementary information and Supplementary Fig. S1).

## Additional Information

**How to cite this article**: Landi, G. *et al*. A noise model for the evaluation of defect states in solar cells. *Sci. Rep.* **6**, 29685; doi: 10.1038/srep29685 (2016).

## Supplementary Material

Supplementary Information

## Figures and Tables

**Table 1:** Relevant parameters, useful for the evaluation of the solar cell degradation process, extracted from dc analysis (open-circuit voltage *V*_*oc*_, short-circuit current *I*_*sc*_, and power conversion efficiency *η*) and from noise measurements (total trap density *N*_*T*_) at a reference temperature of 300 K.

Fluence (*p*^+^/*cm*^2^)	*V*_*oc*_ (*mV*)	*I*_*sc*_ (*mA*)	*η* (%)	*N*_*T*_ (10^15^ *cm*^−3^)
0	(581 ± 6)	(37.4 ± 0.5)	(15.0 ± 0.3)	(8.4 ± 0.4)
2 × 10^11^	(567 ± 5)	(34.2 ± 0.4)	(14.1 ± 0.3)	(20.1 ± 0.9)
1 × 10^12^	(547 ± 5)	(31.7 ± 0.4)	(12.7 ± 0.2)	(28 ± 1)
5 × 10^12^	(518 ± 4)	(27.3 ± 0.3)	(10.3 ± 0.2)	(36 ± 2)
