# Supplementary material for: A noise model for the evaluation of defect states in solar cells
Source: Sci Rep. 2016 Jul 14;6:29685. doi: 10.1038/srep29685 (PMC4944190; doi:10.1038/srep29685)
Supplement: Supplementary Information [file srep29685-s1.pdf]

Supplementary Information for "A noise model for the evaluation of defect states in solar cells"

G. Landi[1],[*] C. Barone[2,3],[†] C. Mauro[2,3], H. C. Neitzert[1], and S. Pagano[2,3]

[1]*Dipartimento di Ingegneria Industriale, Università di Salerno, I-84084 Fisciano, Salerno, Italy*

[2]*Dipartimento di Fisica "E.R. Caianiello", Università di Salerno, I-84084 Fisciano, Salerno, Italy*

[3]*CNR-SPIN, UOS di Salerno, I-84084 Fisciano, Salerno, Italy*

[*]Electronic address: glandi@unisa.it

[†]Electronic address: cbarone@unisa.it

Table S1: Best values of the fitting coefficients I_0^{light}, I_A, A_1, and A_2, from equation (8) in the main text. The case of pristine Si-based solar cells is considered in the temperature range between 280 and 340 K (step 10 K).

Temperature (K)	I_0^{light} (mA)	I_A (mA)	A_1 (A)	A_2 (A)
280	(5.32±0.06)	(261±3)	$(9.9\pm0.1)\times10^{-12}$	$(2.32\pm0.03)\times10^{-13}$
290	(4.13±0.09)	(165±4)	$(1.23\pm0.03)\times10^{-11}$	$(3.83\pm0.08)\times10^{-13}$
300	(3.39±0.05)	(136±2)	$(1.49\pm0.02)\times10^{-11}$	$(4.76\pm0.07)\times10^{-13}$
310	(2.84±0.05)	(112±2)	$(1.74\pm0.03)\times10^{-11}$	$(5.92\pm0.09)\times10^{-13}$
320	(2.40±0.03)	(98±1)	$(2.02\pm0.02)\times10^{-11}$	$(6.91\pm0.07)\times10^{-13}$
330	(2.11±0.05)	(74±2)	$(2.27\pm0.05)\times10^{-11}$	$(8.2\pm0.2)\times10^{-13}$
340	(1.78±0.03)	(45±1)	$(2.65\pm0.04)\times10^{-11}$	$(1.08\pm0.02)\times10^{-12}$

Evaluation of spurious noise contributions

The intrinsic nature of the fluctuation mechanisms, characteristic of the devices investigated, must be first verified by excluding the possible influence of spurious external noise contributions, such as the photon number fluctuation, light source bias circuit noise, and light source emission noise. All these contributions can be evaluated by measuring the voltage-noise spectrum generated in a small size (15 mm^2) silicon photodetector (model "OSD15-5T") placed in the same position of the solar cell under test. The experimental spectral density of the photodetector $S_{V_{pd}}^{exp}$ is expressed as

$$S_{V_{pd}}^{exp} = S_{V_{pd}}^{int} + S_V^{ro} + S_V^{ill} \ , \tag{1}$$

where $S_{V_{pd}}^{int}$ is the intrinsic photodetector noise, S_V^{ro} is the spectral measurement system background noise (negligible as discussed in the main text of the paper), and S_V^{ill} is the noise due to the illumination beam. In the case here considered, S_V^{ill} is dominant, thus allowing to estimate the amount of illumination fluctuations. By simple rescaling, it is possible to compute the contribution of such noise source on the solar cell measured voltage-spectral density as

$$S_{V_{cell}}^{ill} = S_{V_{pd}}^{exp} \cdot \frac{R_{D_{cell}}^2}{R_{D_{pd}}^2} \cdot \frac{E_{cell}^2}{E_{pd}^2} \cdot \frac{A_{cell}}{A_{pd}} \ , \tag{2}$$

where $R_{D_{cell}}$ and $R_{D_{pd}}$ are the dynamical resistances of the solar cell and photodetector at the bias point and under illumination, respectively, $E_{cell} = 0.45$ A/W and $E_{pd} = 0.33$ A/W are the responsivities of the solar cell and photodetector, respectively, A_{cell} and A_{pd} are the areas of the solar cell and photodetector, respectively. In Fig. **S1** the measured overall and illumination noise spectra are shown for different illumination levels and at 300 K. As clearly evident from the figure, the illumination noise is negligible in all cases.

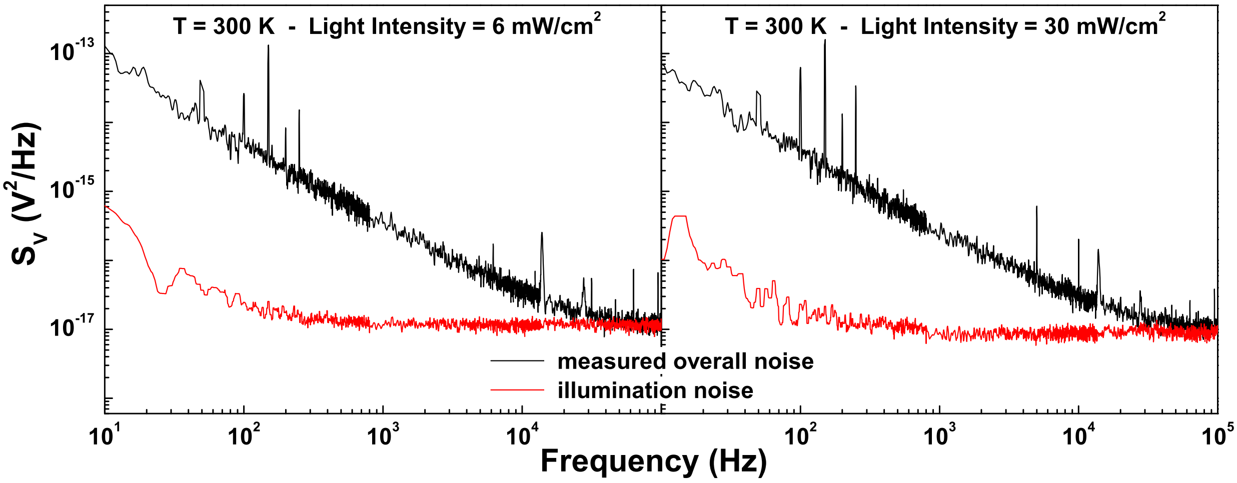

Figure S1: **Intrinsic and external noise contributions.** Frequency dependence of the measured overall noise (black trace) and the illumination noise (red trace), obtained with Eq. (2), at 6 mW/cm^2 (left panel) and 30 mW/cm^2 (right panel).
